# The Spectrum of Kidney Pathology in B-Cell Chronic Lymphocytic Leukemia / Small Lymphocytic Lymphoma: A 25-Year Multicenter Experience

**DOI:** 10.1371/journal.pone.0119156

**Published:** 2015-03-26

**Authors:** Anne-Laure Poitou-Verkinder, Arnaud Francois, Fanny Drieux, Stéphane Lepretre, Bruno Legallicier, Bruno Moulin, Michel Godin, Dominique Guerrot

**Affiliations:** 1 Service de Néphrologie, CHU Hôpitaux de Rouen, Rouen, France; 2 Service d’Anatomie Pathologique, CHU Hôpitaux de Rouen, Rouen, France; 3 Service d’Hématologie, Centre Henri Becquerel, Rouen, France; 4 Service de Néphrologie, CHU Hôpitaux de Strasbourg, Strasbourg, France; 5 INSERM Unité 1096, Université de Médecine-Pharmacie de Rouen, Rouen, France; Fondazione IRCCS Ospedale Maggiore Policlinico & Fondazione D’Amico per la Ricerca sulle Malattie Renali, ITALY

## Abstract

**Background:**

Chronic lymphocytic leukemia and small lymphocytic lymphoma are 2 different presentations of the most common B-cell neoplasm in western countries (CLL/SLL). In this disease, kidney involvement is usually silent, and is rarely reported in the literature. This study provides a clinicopathological analysis of all-cause kidney disease in CLL/SLL patients.

**Methods:**

Fifteen CLL/SLL patients with kidney biopsy were identified retrospectively. Demographic, clinical, pathological and laboratory data were assessed at biopsy, and during follow-up.

**Results:**

At biopsy 11 patients presented impaired renal function, 7 patients nephrotic syndrome, 6 patients dysproteinemia, and 3 patients cryoglobulinemia. Kidney pathology revealed CLL/SLL-specific monoclonal infiltrate in 10 biopsies, glomerulopathy in 9 biopsies (5 membranoproliferative glomerulonephritis, 2 minimal change disease, 1 glomerulonephritis with organized microtubular monoclonal immunoglobulin deposits, 1 AHL amyloidosis). Five patients presented interstitial granulomas attributed to CLL/SLL. After treatment of the hematological disease, improvement of renal function was observed in 7/11 patients, and remission of nephrotic syndrome in 5/7 patients. During follow-up, aggravation of the kidney disease systematically occurred in the absence of favorable response to hematological treatment.

**Conclusions:**

A broad spectrum of kidney diseases is associated with CLL/SLL. In this setting, kidney biopsy can provide important information for diagnosis and therapeutic guidance.

## Introduction

Kidney diseases are frequently associated with hematologic malignancies. Depending on the type and stage of the malignancy, the spectrum of kidney pathology can be particularly wide, including immune-mediated glomerulonephritis, interstitial monoclonal infiltration and tubular obstruction.

Chronic Lymphocytic Leukemia (CLL) and Small Lymphocytic Lymphoma (SLL) are two different presentations of the most frequent B-cell neoplasm in adults in Western countries, with an annual incidence of 4–30 cases per 100,000 persons per year [1,2]. Although most patients remain asymptomatic, the clinical features of CLL/SLL are highly variable, including presentation, course and outcome. Unlike renal involvement in monoclonal gammapathies, the description of kidney diseases associated with CLL/SLL is currently limited to case reports and small series of immune-mediated glomerulopathies and interstitial infiltration by monoclonal lymphocytes [3–9].

In this context, the objective of the present study was to provide a systematic description of kidney pathology and clinical characteristics in a well characterized population of CLL/SLL patients referred for kidney biopsy, in order to better delineate the spectrum and the relative frequency of kidney diseases associated with CLL/SLL.

## Subjects and Methods

### Patients

This study included all the CLL/SLL patients who underwent a kidney biopsy in Haute-Normandie (1 800 000 inhabitants) between 1989 and 2014. During the study period, 3950 kidney biopsies were performed in the 8 hospitals and clinics of the region. All the biopsies were processed and analyzed in a single central reference pathology department, where the samples were systematically included in a tissue bank (Pathology Department, Rouen University Hospital, Rouen, France). Finally, after screening of the central patient database, 15 patients (20 biopsies) were included on the basis of a CLL/SLL diagnosis ascertained before or at the time of the kidney biopsy. Two of the 15 patients were reported in a previous study specifically focusing on glomerulonephritis in CLL/SLL [8]. Demographic, clinical and laboratory data, including results of the nephrological investigations prompting kidney biopsy, were recorded at time of the biopsy, and from the initial to the latest follow-up.

CLL/SLL was diagnosed according to the WHO classification (ICD-0 9823/3) [1,2]. An indicative extrapolation based on data from the Basse-Normandie CLL/SLL registry estimates that approximately 525 new cases of SLL and 2875 of CLL were diagnosed in Haute-Normandie over the 25-year study period. The diagnosis of CLL requires the presence of at least 5.10^9^ B lymphocytes /l in the peripheral blood, over > 3 months, with evidence of monoclonality (CD5, CD19, CD20, CD23) [1,2]. CLL is distinguishable from SLL by its leukemic appearance. The definition of SLL requires a histological analysis of lymph node biopsy, and the clinical presentation includes lymphadenopathy and/or splenomegaly. In this case, lymph node histology shows small lymphocytes with condensed chromatin and, occasionally, small nucleolus. Large lymphoid cells with more prominent nucleoli and dispersed chromatin are typically present, and are usually clustered in pseudofollicles. The Binet staging system classifies CLL in stages A, B, or C according to the number of lymphoid tissues involved (enlarged lymph nodes in the neck, groin, underarms, hepatomegaly or splenomegaly), and to the presence of anemia and thrombocytopenia. Matutes score is based on the most common marker profile in CLL (CD5+, CD23+, FMC7- and weak surface immunoglobulin (SIg) and CD22), and is used to distinguish between typical (Matutes 4 and 5) and atypical CLL, by assigning scores that range from 5 to 0 [10]. General signs were defined as A: no general sign; or B: weight loss > 10% in 6 months, and/or fever > 38°C without alternative diagnosis and/or nocturnal sweating. Tumoral syndrome was defined as the presence of lymphadenopathy and/or extrarenal organ infiltration (hepatomegaly, splenomegaly). Hematological response to treatment was defined according to the reference guidelines [1]. Dysproteinemias were identified by serum protein immunoelectrophoresis and immunofixation.

Nephrotic syndrome (NS) was defined as proteinuria > 3 g/d with albuminemia < 30 g/l. Complete remission was defined as proteinuria < 0.3 g/d, partial remission as proteinuria < 3 g/d, and relapse as proteinuria > 3g/d after initial remission [11]. Acute renal failure was defined by an increase in plasma creatinine > 26.5 micromol/l and/or >150% baseline value, according to the Acute Kidney Injury Network (AKIN) criteria [12]. Kidney function improvement was defined as a decrease in plasma creatinine > 10%, with persistence > 3 months. Chronic kidney disease was defined as a decrease in MRDR eGFR < 90 ml/min/1.73 m² with persistence > 3 months [13].

### Kidney pathology

The 20 biopsies were processed in a standardized manner in Rouen pathology department. Light microscopy analysis was performed after fixation in Dubosq Brazil solution, 2-μm paraffin section, and coloration by Masson trichrome, hematoxylin & eosin, Marinozzi silver, and Congo red stainings. Standardized immunofluorescence was performed retrospectively in 2014. 3-μm cryostat sections were incubated with FITC-conjugated antibodies to human Ig gamma, alpha, mu, kappa, and lambda light chains, C3, C4, C1q, fibrinogen, albumin, CD5, CD20, CD23, CD3, Cyclin D1 and CD10. The biopsies were reanalyzed in a blinded fashion by 2 experienced kidney and hematology pathologists. Ultrastructural electron microscopy was performed in 7 patients.

### Ethical requirements

This study was in accordance with the Helsinki declaration, and was approved by the local ethics board for non-invasive health research (Comité d'Ethique pour la Recherche Non Interventionnelle CERNI N°E-2014-11, for the Centre de Protection des Personnes Nord-Ouest-I, Rouen University Hospital, Rouen, France), which waived the need for informed consent in this retrospective analysis.

## Results

### Clinical presentation at kidney biopsy

The clinical features at biopsy are presented in Table 1. The CLL/SLL had been diagnosed before the nephropathy in 7/15 cases, with a mean interval of 61 months from the hematological to the nephrological diagnosis in this setting (range 19–156 months). General symptoms were present in 7/15 patients, and 11/15 patients presented lymphadenopathy, hepatomegaly, and/or splenomegaly. Extrarenal symptoms were present in 4/15 patients (peripheral neuropathy, arthralgia, skin vasculitis, cholestasis).

**Table 1:** Clinical characteristics at biopsy.

Case	Sex	Age at biopsy	Interval CLL/SLL Nephropathy (m)	CLL/SLL	Hematological treatment before biopsy	Comorbidity	HTN	Extracellular fluid expansion	Extrarenal symptoms	General symptoms A/B	Tumoral syndrome
*Interstitial nephropathy*: *isolated specific tumoral interstitial infiltrate +/- CLL/SLL-related granulomatosis*											
1	M	65	36 p	CLL	N	HTN, smoking, dyslipidemia, spondylarthropathy	Y	N	N	A	Y
2	M	69	19	CLL	Cs	HTN, smoking, T2D, stroke, alcoholism	Y	Y	N	B	Y
3	F	51	36	CLL	R-FC	HTA	Y	Y	N	A	Y
4	M	57	96	CLL	N	Smoking, peripheral artery disease	Y	N	N	A	Y
5	M	77	62	CLL	R-FC	HTN, smoking, sleep apnoea syndrome	Y	N	N	B	Y
5bis		78			R-FC + Chlorambucil		Y	N	N	A	Y
6	M	67	0	CLL	N	Smoking	Y	N	N	B	N
*Glomerular lesions associated or not with interstitial nephropathy*											
7	M	73	156	CLL	N	HTN, smoking	Y	Y	N	A	N
8	F	57	42	CLL	N	Preeclampsia	N	Y	N	A	N
8bis		60			Cs		Y	Y	N	A	N
9	M	62	0	SLL	N	HTN	Y	Y	N	B	Y
10	F	67	19 p	CLL	N	N	Y	Y	Y	A	N
10bis		69			N		Y	Y	Y	B	N
11	F	65	0	SLL	N	Dyslipidemia	Y	Y	Y	A	Y
12	F	63	19	CLL	Chlorambucil	T2D, peripheral artery disease	Y	Y	Y	A	Y
12bis		71			Chlorambucil		Y	Y	N	B	N
13	M	76	0	SLL	N	N	Y	Y	N	B	Y
14	M	62	0	CLL	N	Dilated cardiomyopathy	N	Y	Y	B	Y
15	F	67	0	CLL	N	N	Y	Y	N	B	Y
15bis		78			Chlorambucil + Fludarabine		Y	Y	N	A	Y

p, CLL/SLL diagnosis post-nephropathy; Cs, corticosteroids; R-FC, rituximab+fludarabine+cyclophosphamide; HTN, hypertension; T2D, type 2 diabetes; Y, yes; N, no.

### Laboratory and radiological data

In all patients, the kidney biopsy was performed because of renal failure and/or significant proteinuria. The laboratory and radiological results are presented in Tables 2 and 3. MDRD eGFR < 60 ml/min/1.73m² was found in 11/15 patients, (mean 23 ml/min/1.73m², range 7–38). NS was present in 7/15 patients, while normal proteinuria and urine sediment were found in 2 cases. The hematological work-up revealed dysproteinemia in 6/15 patients (cases 4, 5, 10, 11, 12, 14), with cryoglobulinemia in 3 cases (cases 10, 11, 12). Complement activation was found in 5/15 patients (cases 9, 10, 11, 12, 13). Enlarged kidneys were noticed in one patient (case 6).

**Table 2:** Laboratory and radiological features.

Case	AKI/ CKD	P. creatinine (micromol/l) MDRD eGFR (ml/min)	Proteinuria (g/d) P. albumin (g/l)	Hematuria	Leukocyturia	Dysproteinemia	Fanconi Syndrome	Complement	Kidney length (R/L, mm)
*Interstitial nephropathy*: *isolated specific tumoral interstitial infiltrate +/- CLL/SLL-related granulomatosis*									
1	CKD	185 / 34	0.3 / 56	N	N	N	N	Normal	101 / 110
2	AKI	743 / 7	1 / 39	Y	Y	N	N	Normal	120 / 120
3	CKD	178 / 28	0.9 / 43	N	Y	N	N	Normal	120 / 120
4	CKD	194 / 36	0.3 / 49	N	N	IgG kappa 4.6 g/l / kappa BJ	Y	Normal	120 / 107
5	CKD	247 / 24	0.8 / 44	N	N	Lambda BJ	N	Normal	113 / 113
5bis	AKI/CKD	638 / 8	1.4 / 41	N	N	N	Y	Normal	Normal
6	AKI	616 / 8	1.2 / 36	Y	Y	N	N	Normal	>120 / >120
*Glomerular lesions associated or not with interstitial nephropathy*									
7	CKD	193 / 38	3.8 / 44	Y	N	N	N	Normal	118 / 108
8	N	74 / 72	6.1 / 20	N	N	N	N	Normal	Normal
8bis	N	71 / 76	4.6 / 32	Y	N	IgG lambda	N	Normal	Normal
9	AKI	178 / 36	6.3 / 21	Y	N	N	N	Low C3	Normal
10	N	71 / 75	1.2 / 43	Y	Y	Type II cryoglobulin	N	Low C3 and C4	117 / 112
10bis	CKD	96 / 53	13 / 19	Y	Y	Type II cryoglobulin	N	Low C3 and C4	ND
11	CKD	168 / 28	14 / 25	Y	Y	Type I cryoglobulin, IgG kappa	N	Low C3 and C4	103 / 108
12	AKI/CKD	536 / 7	>3 / 30	Y	Y	Type I cryoglobulin, IgG kappa	N	Low C4	118 / 123
12bis	AKI/CKD	769 / 5	4.5 / 25	Y	Y	IgG kappa	N	Normal	95 / 95
13	N	100 / 67	>6 / 24	Y	Y	N	N	Low C3 and C4	Normal
14	CKD	470 / 12	7 / 24	N	N	IgG lambda	N	ND	116 / 100
15	N	79 / 67	6.1 / 30	Y	Y	N	N	Normal	Normal
15bis	CKD	530 / 7	>13 / 28	Y	Y	N	N	Normal	Normal

AKI, acute kidney injury; CKD, chronic kidney disease; P, plasma; BJ, Bence Jones proteinuria; ND, no data available.

**Table 3:** Laboratory and radiological features.

Case	Lymphocyte count (/mm3)	Cytopenia (0;1;2)	Blood flow cytometry	Matutes	Bone marrow lymphocyte infiltration	Binet stage
*Interstitial nephropathy*: *isolated specific tumoral interstitial infiltrate +/- CLL/SLL-related granulomatosis*						
1	16000	0	Lambda, CD5+ CD19+ CD23+ CD38- SmIg strong	2	76%	A
2	70680	2 (Hb,platelets)	Lambda, CD5+ CD19+	ND	62%	C
3	1380	0	Kappa, CD5+ CD19+ CD23+ CD38-	5	ND	B
4	102000	1 (platelets)	CD5+ CD19+ CD23+ FMC7- SmIg weak	5	35%	B
5	102300	1 (platelets)	Lambda, CD5+ CD19+ CD23+ FMC7- CD38-	5	ND	C
5bis	7605	2 (Hb, platelets)		5	ND	C
6	80170	1 (Hb)	CD5+ CD19+ CD23+ FMC7- CD38- CD79b weak SmIg weak	5	ND	A
*Glomerular lesions associated or not with interstitial nephropathy*						
7	27200	0	Kappa, CD5+ CD19+ CD23+	5	ND	A
8	36800	0	Lambda, CD5+ CD19+ SmIg weak	ND	75%	A
8bis	8100	0		ND	ND	A
9	3652	1 (Hb)	CD5+ CD19+ CD23+ CD38+ SmIg strong	/	ND	/
10	3250	0	Kappa, CD5+ CD19+	ND	74%	A
10bis	6960	1 (Hb)	Kappa, CD5+ CD19+	ND	ND	ND
11	7691	1 (Hb)	Kappa, CD5+ CD19+ CD23- FMC7+ CD79b+ weak SmIg weak	/	82%	/
12	7100	0	Kappa, CD5+ CD19+ CD23 weak FMC7+	ND	75%	B
12bis	11590	2 (Hb, platelets)		ND	85%	C
13	10350	1 (Hb: AIHA)	ND	/	78%	/
14	7636	0	Lambda, CD5- CD19- SmIg strong	ND	37%	A
15	9870	1 (Hb)	Lambda, CD5+ CD19+ CD23+ SmIg weak	5	49%	C
15bis	1110	1 (platelets)		5	55%	C

AIHA, autoimmune hemolytic anemia; SmIg, surface membrane immunoglobulin; Hb, hemoglobin; ND, no data available.

### Pathology

**Fig 1:**
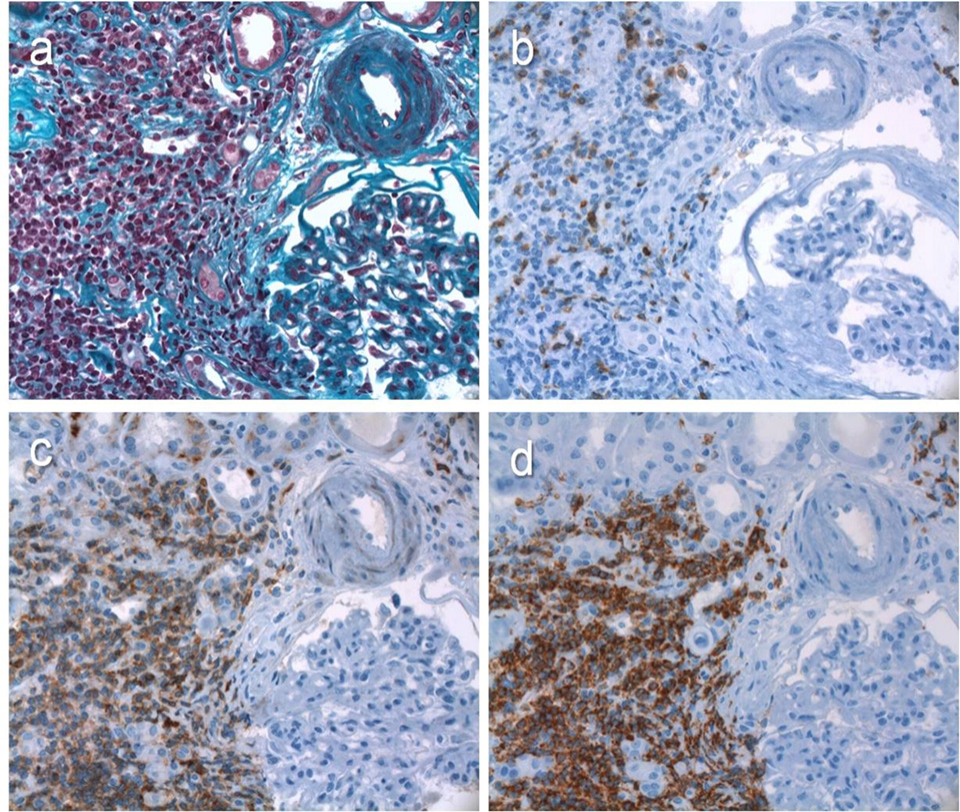
CLL/SLL infiltrate (case 12). (a) Masson trichrome staining. (b) Specific immunostaining to human CD3. (c) Specific immunostaining to human CD5. (d) Specific immunostaining to human CD20. Light microscopy, original magnification, X 40.

Tables 4 and 5 summarize the conclusions of the kidney biopsy analyses. Monoclonal infiltration was evidenced in 10/15 biopsies. All of these 10 patients presented specific and abundant tumoral infiltration, which was generally associated with non specific reactive lymphocytes. The common morphological feature in these cases was a diffuse nodular infiltration, composed of small mature lymphocytes, with small hyperchromatic nuclei, expanding the interstitium at the expense of the tubular structures, the peritubular capillaries, and less frequently the glomeruli (Fig. 1). Immunohistochemistry revealed monotypic lymphocytes (CD20+, CD5+, Cyclin D1-, CD10-). Six patients presented interstitial infiltration without glomerular lesions. Four had a severe infiltration, associated with GFR < 30 ml/min/1.73m², which represented the indication for kidney biopsy. There was no clear association between the intensity of interstitial infiltration, the CLL stage, and the acute *vs* chronic presentation of kidney failure. A tumoral syndrome was found in 9/10 of the patients with interstitial infiltration, compared to 2/5 patients without interstitial infiltration. Acute kidney injury was attributed to tubular necrosis in 2 patients (cases 6 and 9), and to interstitial infiltration in cases 2, 5, and 12. In the patients with AKI occurring in a context of MPGN, no extracapillary proliferation was found.

**Table 4:** Kidney biopsy pathology.

Case	Light microscopy	Glomerular immunofluorescence	Infiltrate immunohistochemistry	Electron microscopy	Definite diagnosis
*Interstitial nephropathy*: *isolated specific tumoral interstitial infiltrate +/- CLL/SLL-related granulomatosis*					
1	Mild monomorphic diffuse infiltrate	Ig alpha Ig mu traces	CD20+ CD5+ CD23- Cyclin D1- CD10-		CLL infiltrate
2	Moderate monomorphic diffuse infiltrate	Normal	CD20+ CD5+ CD23- CD10- Cyclin D1-		CLL infiltrate
3	Moderate monomorphic focal infiltrate	Ig gamma Ig mu C3 C1q traces	CD20+ CD5+ CD23- CD10- Cyclin D1-		CLL infiltrate
4	Two monomorphic nodules	Normal	CD20+ CD5+ CD23- CD79a weak CD10- (nodules)		CLL infiltrate Granulomatous reaction
	Abundant interstitial granulomas				
	Mild polymorphic diffuse infiltrate				
5	One interstitial granuloma	ND	CD20+ CD5+ CD23- CD10- Cyclin D1-		CLL infiltrate Granulomatous reaction
	Severe monomorphic diffuse infiltrate				
5bis	Abundant interstitial granulomas	Normal	CD20+ CD5+ CD23- CD10-		CLL infiltrate Granulomatous reaction
	Severe monomorphic diffuse infiltrate				
6	Abundant epithelioid non-necrotic peritubular granulomas	Normal	CD20+ CD5+ CD79a+ CD23- CD10- Cyclin D1- CD1-		CLL infiltrate Granulomatous reaction
	Severe monomorphic diffuse infiltrate				
*Glomerular lesions associated or not to interstitial nephropathy*					
7	MCD	Ig mu C3 C4 C1q traces	CD20- CD3+ CD5+ CD19- CD23- Cyclin D1-		MCD
	Mild polymorphic diffuse infiltrate				
8	MCD	ND	/	Foot process fusion	MCD
8bis	MCD	Normal	/		MCD
9	MPGN with capillary thrombi	Ig gamma Ig mu kappa lambda C4 C1q, C3 traces	CD20+ CD5- CD23- Cyclin D1- (nodule)	Abundant subendothelial non-organized non-fibrillar deposits	MPGN
	One monomorphic nodule, mild polymorphic infiltrate				
10	MPGN with endocapillary proliferation	ND	/	Rare non-oriented cylindric 280-nm long 35-nm diameter deposits	Cryoglobulin-related MPGN
10bis	MPGN with endocapillary proliferation and fibrinoid deposits	Ig gamma Ig alpha Ig mu C1q C3 C4	/		Cryoglobulin-related MPGN
	Pseudo-thrombi				
11	MPGN with endo- and extra-capillary proliferation	Ig gamma1 kappa C3, C1q traces	CD20+ CD5+ CD23+ CD10-	Subendothelial granular and fibrillar deposits	Cryoglobulin-related MPGN CLL infiltrate
	Pseudo-thrombi			No fibrillary organization in lymphocytes	
	Severe monomorphic diffuse infiltrate				
12	MPGN Pseudo-thrombi	Ig gamma kappa C3	CD20+ CD5+ CD23- CD10- Cyclin D1-		Cryoglobulin-related MPGN CLL infiltrate
	Moderate monomorphic diffuse infiltrate				
12bis	MPGN Pseudo-thrombi	Ig gamma kappa C3	CD20+ CD5+ CD23- CD10- Cyclin D1-		Cryoglobulin-related MPGN CLL infiltrate
	Severe monomorphic diffuse infiltrate				
13	MPGN appearance	Ig gamma kappa C3	ND	Endomembranous non-amyloid microfibrillar 8-nm diameter deposits	Fibrillary glomerulonephritis CLL infiltrate
	Moderate monomorphic focal infiltrate				
14	Amyloidosis	Ig gamma lambda	CD20+ CD5- CD23- CD10- Cyclin D1-	Non-oriented multifocal 10-nm-large fibers	AHL Amyloidosis Granulomatous reaction
	Mild polymorphic infiltrate				
	One interstitial granuloma				
15	Mesangial sclerosis	Ig gamma lambda C3	CD20+ CD5+ CD23- Cyclin D1-	Subendothelial organized microtubular deposits	ITGN
	One interstitial granuloma			30-nm long external diameter	CLL infiltrate
	Mild monomorphic focal infiltrate				Granulomatous reaction
15bis	Mesangial sclerosis and proliferation, capillary deposits, podocytosis	ND	CD20+ CD5+ CD23- CD10- Cyclin D1-		ITGN
	Moderate polymorphic multifocal infiltrate				CLL infiltrate

MPGN, membranoproliferative glomerulonephritis; AHL, heavy- and light-chain amyloidosis; ITGN, immunotactoid/microtubular glomerulonephritis; ND, no data available

MCD, minimal change disease; MPGN, membranoproliferative glomerulonephritis; ND, no data available.

**Table 5:** Kidney biopsy pathology.

Case	Light microscopy	Glomerular immunofluorescence	Infiltrate immunohistochemistry	Electron microscopy	Definite diagnosis
11	MPGN with endo- and extra-capillary proliferation; Pseudo-thrombi; Severe monomorphic diffuse infiltrate	Ig gamma1 kappa C3, C1q traces	CD20+ CD5+ CD23+ CD10-	Subendothelial granular and fibrillar deposits; No fibrillary organization in lymphocytes	Cryoglobulin-related MPGN CLL infiltrate
12	MPGN; Pseudo-thrombi; Moderate monomorphic diffuse infiltrate	Ig gamma kappa C3	CD20+ CD5+ CD23- CD10- Cyclin D1-		Cryoglobulin-related MPGN CLL infiltrate
12bis	MPGN; Pseudo-thrombi; Severe monomorphic diffuse infiltrate	Ig gamma kappa C3	CD20+ CD5+ CD23- CD10- Cyclin D1-		Cryoglobulin-related MPGN CLL infiltrate
13	MPGN appearance; Moderate monomorphic focal infiltrate	Ig gamma kappa C3	ND	Endomembranous non-amyloid microfibrillar 8-nm diameter deposits	Fibrillary glomerulonephritis CLL infiltrate
14	Amyloidosis; Mild polymorphic infiltrate; One interstitial granuloma	Ig gamma lambda	CD20+ CD5- CD23- CD10- Cyclin D1-	Non-oriented multifocal 10-nm-large fibers	AHL Amyloidosis Granulomatous reaction
15	Mesangial sclerosis; One interstitial granuloma; Mild monomorphic focal infiltrate	Ig gamma lambda C3	CD20+ CD5+ CD23- Cyclin D1-	Subendothelial organized microtubular deposits; 30-nm long external diameter	ITGN; CLL infiltrate Granulomatous reaction
15bis	Mesangial sclerosis and proliferation; Capillary deposits; Podocytosis; Moderate polymorphic multifocal infiltrate	ND	CD20+ CD5+ CD23- CD10- Cyclin D1-		ITGN; CLL infiltrate

MPGN, membranoproliferative glomerulonephritis; AHL, heavy- and light-chain amyloidosis; ITGN, immunotactoid/microtubular glomerulonephritis; ND, no data available.

**Fig 2:**
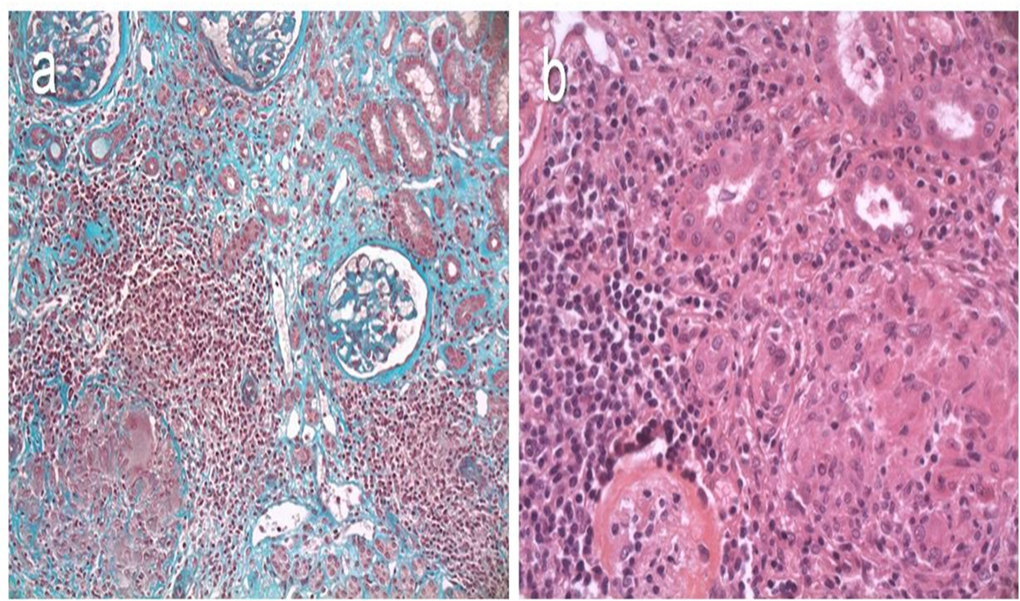
Representative cases of interstitial granulomas. (a) Case 4. Masson trichrome. Interstitial epithelioid and gigantocellular non-necrotizing granuloma, surrounded by lymphocytes. Original magnification, X 20. (b) Case 6. Hematoxylin and eosin staining. Granuloma associated with severe diffuse CLL/SLL monomorphic infiltrate. Original magnification, X 40.

Interstitial granulomas were found in 5/15 patients. Light microscopy revealed florid interstitial gigantocellular granulomas, surrounded by lymphocytes (Fig. 2). No tubular basement membrane rupture, cellular necrosis or microcristalline precipitation was observed, and Periodic Acid Schiff, Ziehl and Grocott stainings revealed no microorganism. Immunofluorescence showed no specific interstitial deposit. Granulomas were a prominent feature in cases 4, 6 and on the 2^nd^ biopsy of case 8. The granulomas were associated with monotypic interstitial infiltration (cases 4, 5, 6), or with glomerular deposits (cases 14, 15). No extrarenal granuloma was found in these patients.

**Fig 3:**
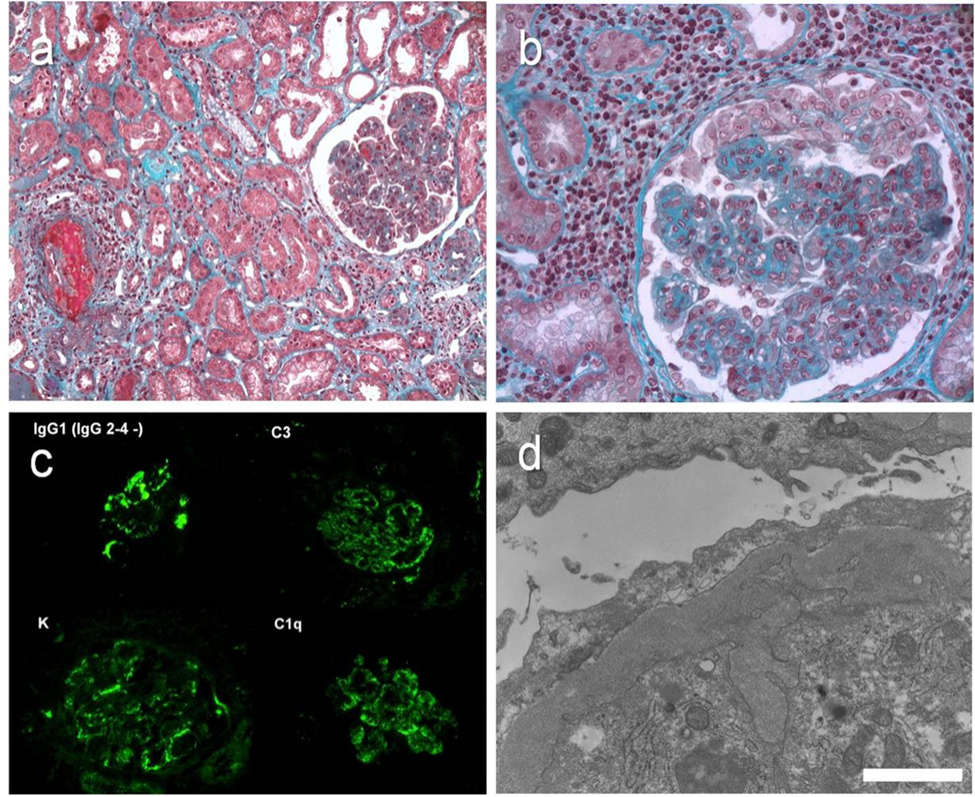
Representative cases of MPGN. (a) Case 10. Light microscopy, Masson trichrome staining. Cryoglobulin-related MPGN. Endocapillary proliferation, fibrinoid necrosis and pseudo-thrombi. Original magnification, X 20. (b) Case 11. Light microscopy, Masson trichrome. Endo- and extracapillary proliferation. Note the severe CLL/SLL monomorphic infiltrate. Original magnification, X 40. (c) Case 11 (IgG kappa type I cryoglobulin). Immunofluorescence microscopy. Endomembranous deposits of IgG1, kappa, C3, C1q. Original magnification, X 20. (d) Case 11. Electron microscopy. Subendothelial granular and fibrillar deposits. Bar = 1200 nm.

Nine patients presented glomerular lesions, of which 4/9 were associated with monoclonal interstitial infiltration. Membranoproliferative glomerulonephritis (MPGN) was present in 5 patients (cases 9–13). Light microscopy revealed typical mesangial and/or endocapillary proliferation, double contours of the basement membrane, and endomembranous deposits (Fig. 3). The deposits were related to a circulating cryoglobulin in cases 10–12. In these 3 patients an important polymorphic infiltration with polymorphonuclear cells, lymphocytes and macrophages associated with endomembranous deposits was responsible for the formation of pseudothrombi in capillary loops, and fibrinoid deposits in one patient (case 10). The ultrastructural study revealed proliferative mesangial cells, podocyte foot process fusion, and endomembranous homogeneous granular deposits. The immunofluorescence confirmed the presence of the circulating cryoglobulin within glomerular deposits. Hepatitis B, C, and HIV serologies were negative in all patients. In patients 9 and 13 neither cryoglobulin nor dysproteinemia were detected. Case 13 presented monotypic gamma and kappa deposits together with positive C3 on immunofluorescence analysis, and decreased C3 and C4 in the peripheral blood tests. Large endomembranous deposits characterized by 8-nm diameter microfibrillar structures, organized in 0.96-μm long and 0.45-μm large bundles composed of approximately 12 elements were evidenced, leading to the diagnosis of fibrillary glomerulonephritis.

**Fig 4:**
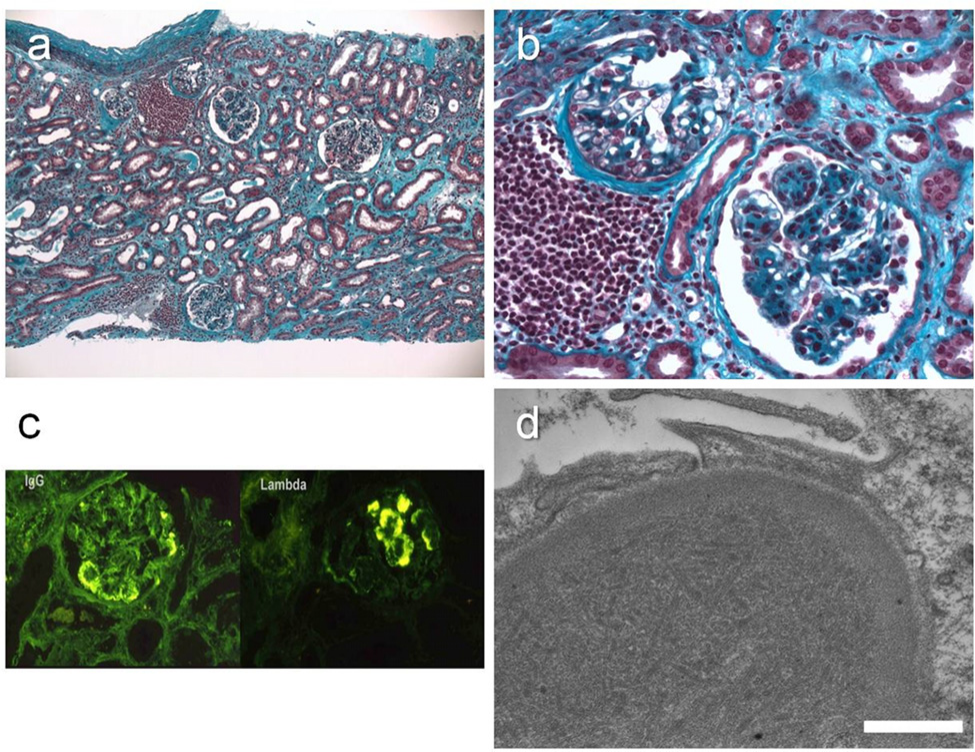
GOMMID (case 15) (a) Light microscopy. Masson trichrome. Mesangial sclerosis with focal mesangial proliferation. Note mild monomorphic focal CLL/SLL infiltrate. Original magnification, X 10. (b) Light microscopy. Masson trichrome. Mesangial sclerosis and proliferation, capillary deposits. Original magnification, X 40. (c) Immunofluorescence microscopy. Monotypic Ig lambda deposits. The same fluorescence patern was obtained with an anti-C3 antibody (data not shown). (d) Electron microscopy. Subendothelial organized microtubular deposits, 30-nm external diameter. Bar = 1200 nm.

Patient 15 presented an immunotactoid glomerulonephritis with organized microtubular monoclonal immunoglobulin deposits. Light microscopy revealed mesangial sclerosis with focal mesangial proliferation (Fig. 4). Large globular deposits were observed in the capillary loops. Monotypic IgG lambda was found within the deposits, with endocapillary C3. The ultrastructural analysis revealed subendothelial organized microtubular deposits, characterized by a 30-nm long external diameter.

Two patients presented minimal change lesions, with normal light microscopy, normal immunofluorescence, and foot process effacement on the ultrastructural analysis (cases 7, 8).

**Fig 5:**
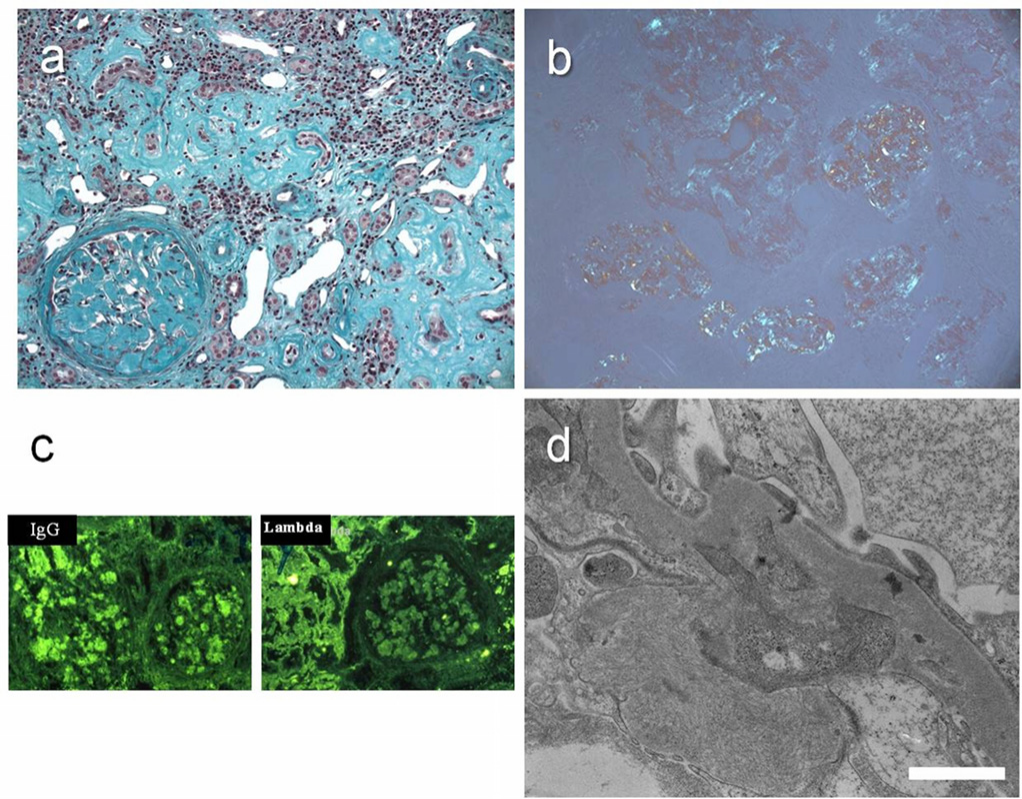
AHL amyloidosis (case 14) (a) Light microscopy. Masson trichrome staining. Massive amorphous deposits in the mesangium and extending to the subendothelial space of glomerular capillaries, Bowman’s capsule, the interstitium, the tubular basement membrane and the arterioles. Original magnification, X 20. (b) Apple green birefringence detected under polarized light. Original magnification, X 20. (c) Immunofluorescence microscopy. Monotypic Ig lambda deposits. Equally intense staining for heavy and light chains. (d) Electron microscopy. Non oriented, randomly arranged, 10-nm diameter fibrils. Bar = 1250 nm.

Patient 14 had glomerular heavy- and light-chain amyloidosis (AHL) related to a circulating IgG lambda monoclonal protein. Light microscopy found very large Congo red positive dichroic deposits in the mesangium, the subendothelial space of glomerular capillaries, Bowman’s capsule, the interstitium, the tubular basement membrane and the arterioles (Fig. 5). Immunofluorescence revealed an equally intense staining for gamma and lambda chains, and ultrastructural analysis showed that the deposits were composed by non-oriented 10-nm large fibers.

### Evolution

The mean post-biopsy follow-up was 86 months (range 3–199 months) (Tables 6 and 7). All but 1 patient (case 7) received corticosteroids and/or cytotoxic treatment. Two patients with severe interstitial infiltration received methylprednisolone pulses followed by cytotoxic chemotherapy, with favorable renal outcomes (cases 2, 6).

**Table 6:** Post-biopsy follow-up and clinical course.

Case	Treatment	Pcreat / MDRD eGFR before/post treatment	Proteinuria (g/d)	NS	Histology	Dialysis	CLL/SLL	Follow-up	Cause of death
			before/post treatment					Alive / Deceased	
*Interstitial nephropathy*: *isolated specific tumoral interstitial infiltrate +/- CLL/SLL-related granulomatosis*									
1	Cs	185/81	0.3 / < 0.3	/	N	N	Stability	8.3 y Deceased	Acute alcoholic hepatitis
									Sepsis
2	CHOP Cs	743/173	1 / 0.9	/	N	N	Stability	3 m Deceased	ND
3	R-FC	178/147	0.9 / 0.7	/	N	N	CR	5 y Alive	/
4	Chlorambucil	194/180	0.3 / 0.3	/	N	N	PR	18 m Alive	/
5	Chlorambucil	247/609	0.8 / 1.3	/	CLL infiltrate Granulomatous reaction	N	Stability	4 y Alive	/
5bis	N	/	/	/	N	Y	Progression		
6	R-Bendamustine Cs	616/296	1.2 / < 0.3	/	N	Transient hemodialysis (1 m)	CR	3 m Alive	/
*Glomerular lesions associated or not to interstitial nephropathy*									
7	N	/	/	Persistent NS	N	Y	Stability	11 y Deceased	Cardiovascular disease Sepsis
8	Cs	74/91	6.1 / 0.13	CR and relapse	MCD	N	PR	16.5 y Alive	/
8bis	Chlorambucil Cs	71/79	4.6 / <0.3	CR	N	N	CR		
9	R-Bendamustine	178/85	6.3 / 1.4	PR	N	N	PR	6 m Alive	/
10	N	/	/	/	Cryoglobulin-related MPGN	N	Progression	9 y Alive	/
10bis	Cyclophosphamide Cs	96/44	13 / <0.3	CR	N	N	CR		

Cs: corticosteroids; CHOP: cyclophosphamide+ adriamycin+ vincristine+ prednisone; R-FC: rituximab+ fludarabine+ cyclophosphamide; R-Bendamustine: rituximab+ bendamustine; Pcreat: Plasma creatinine (micromol/l); NS: nephrotic syndrome; CR: complete remission; PR: partial remission; ESRD: End Stage Renal Disease; MCD: minimal change disease; Y, yes; N, no; ND, no data available.

**Table 7:** Post-biopsy follow-up and clinical course.

Case	Treatment	Pcreat / MDRD eGFR before/post treatment	Proteinuria (g/d)	NS	Histology	Dialysis	CLL/SLL	Follow-up	Cause of death
			before/post treatment					Alive / Deceased	
11	Chlorambucil Chlorambucil; Rituximab	168/66; 83/74	14 / 0.19; 5.1/ <0.3	CR and relapse*; CR	N	N	Remission and relapse*; CR	9 y Alive	/
12	Chlorambucil	536/180	> 3 / 0.15	CR and relapse*	Cryoglobulin-related MPGN	N	Remission and relapse*	8 y Deceased	Febrile medullary aplasia Acutisation CLL/ ESRD
					CLL infiltrate				
12bis	Chlorambucil Cs	769/790 Dialysis	4.5 / Dialysis	Persistent	N	Y	Progression		
	CHOP								
13	Chlorambucil	100/70	> 6 / <0.3	CR	N	N	CR	9 y Deceased	ND
14	Alkeran Cs	470/700 Dialysis	7 / Dialysis	Persistent	N	Y	Stability	9 y Deceased	ND
15	Chlorambucil; Chlorambucil; Fludarabine; Chlorambucil	79/85	6.1 / < 0.3	CR and relapse; CR and relapse*; CR and relapse*; Persistent NS	ITGN CLL infiltrate	N	Remission; Relapse*; Relapse*; Progression	16 y Alive	/
15bis	Rituximab	530/300 Dialysis	> 13 / Dialysis	PR	N	Y	Stability		

Cs: corticosteroids; CHOP: cyclophosphamide+ adriamycin+ vincristine+ prednisone; R-FC: rituximab+ fludarabine+ cyclophosphamide; R-Bendamustine: rituximab+ bendamustine; Pcreat: Plasma creatinine (micromol/l); NS: nephrotic syndrome; CR: complete remission; PR: partial remission; ESRD: End Stage Renal Disease; MCD: minimal change disease; ITGN, immunotactoid/microtubular glomerulonephritis; Y; yes; N, no; ND, no data available.

*: Simultaneous relapses in NS and CLL/SLL

Among the 7 patients presenting with NS and treated with cytotoxic agents, transient or persistent complete remission was observed in 5 patients. Partial remission was observed in patient 9 (MPGN), while patient 14 (AHL amyloidosis) had a persistent NS. In 4 patients with NS relapse observed during the follow-up, progression of the hematological malignancy was also present. In 7/11 patients presenting with decreased eGFR at biopsy the eGFR increased after treatment. Four patients had a negative decline of eGFR after treatment, which occurred in the absence of hematological response (cases 5, 12bis, 14, 15bis). Five patients had a second kidney biopsy for relapse of NS or rapid decrease in eGFR. In all cases, the second kidney biopsy revealed progression of the renal lesions initially identified, with superimposed or increased interstitial infiltration in cases 5, 12, and 15.

At the last follow-up, progression of the kidney disease had required extrarenal suppleance therapy in 5 patients. Patient 6 needed transient hemodialysis, which was withdrawn after post-treatment recovery of kidney function. The mean plasma creatinine at the end of the follow-up, excluding the 5 patients requiring dialysis, was 122 micromol/l.

## Discussion

This study attempted to better describe the spectrum of kidney pathology associated with CLL/SLL. The first occurrence of kidney disease in CLL was described in 1957 by Scott et al, in a patient presenting with NS [14]. Subsequently, case reports and small series have underlined the diversity of lesions which may be found in CLL/SLL, including interstitial infiltration, immune-mediated glomerulonephritis, and drug toxicity. With the inclusion of 15 patients, the present study provides the largest series of all-cause kidney disease in CLL/SLL currently reported (Table 8). CLL is usually diagnosed in patients > 55 y (90%), with a sex ratio of 1.5 [15]. In this study the patients had a mean age 65.2 at the first kidney biopsy, younger than in the CLL/SLL general population (mean age 72), suggesting that complications due to kidney involvement can be diagnostic in CLL/SLL. Alternatively, patients with kidney disease could also present with more aggressive forms of CLL/SLL.

**Table 8:** Previously reported series.

Reference	Patients	Tumoral	MPGN	MCD	Amyloidosis	Other proliferative GN	Interstitial granuloma	MN	Fibrillary GN	ITGN	MIDD	FSGS
	n	infiltrate										
Present study	15	10	5	2	1		5		1	1		
Moulin et al. 1992 ^3^	13	6	8			1		1	2		1	1
Kowalewska et al. 2011 ^32^	7	7	2	2		1		1				1
Seney et al. 1986	4			2								
Dabbs et al. 1986 ^33^	12		1	2		5						
Da'as et al. 2001 ^10^	3	1						1				
Hill et al. 2002	3		2						1			
Barbour et al. 2011	2	1									2	
Cameron et al. 1974	2					1		1				
Audart et al. 2008	2		1		1							
Sethi et al. 2010	2		2									
Sanchorawala et al. 2006 ^37^	2				2							
Nasr et al. 2012	3									3		
Kourelis et al. 2013 ^39^	4				4							
**TOTAL**	**74**	**25**	**21**	**8**	**8**	**8**	**5**	**4**	**4**	**4**	**3**	**2**

MCD, minimal change disease; FSGS, focal segmental glomerulosclerosis; MN, membranous nephropathy; MPGN, membranoproliferative glomerulonephritis; GN, glomerulonephritis;

MIDD, monoclonal immunoglobulin deposition disease; DN, diabetic nephropathy; ITGN, immunotactoid/microtubular glomerulonephritis.

Single case reports published and not included in these case series are not presented in the Table

Renal interstitial infiltration is a frequent finding in autopsy series of CLL patients, estimated between 44 and 90% of patients. The B-cell infiltrate can either be nodular or diffuse, and its localization classically begins in the subcapsular cortex, at the corticomedullar junction, and along the vasa recta [16,17]. In our patients presenting with isolated infiltrates the lymphocytes were monotypic and presented a monomorphic, regular, and mature aspect, separating and directly infiltrating the tubular epithelium, surrounded by local accumulation of extracellular matrix. Usually, the infiltration of the kidneys by monoclonal B cells is bilateral, asymptomatic, and is diagnosed or suspected in late stages of the disease [18]. Even a massive infiltration of both kidneys can remain totally asymptomatic [5]. Urine sediment is usually normal, and proteinuria < 1 g/d. On the other hand, kidney infiltration can present as acute kidney failure or moderate chronic kidney disease [6,7,19,20]. In our study, although 10 patients presented a CLL/SLL-related infiltrate on kidney biopsy, only one patient had a typical enlargement of kidneys. The intensity of interstitial infiltration is usually correlated with the severity of kidney failure, as suggested by the cases of several patients in our study (cases 2, 3, 5, 6) [16,21]. The mechanisms whereby the infiltrate may contribute to decrease GFR are not univocal, and in particular not limited to a mechanical compression [22]. Proinflammatory and profibrotic cytokines released by the infiltrating and/or resident cells could play an important role in tubulo-interstitial injury [23,24]. Accordingly, observational studies have shown that interstitial fibrosis was more frequent in areas of interstitial infiltration [16,21]. We found no clear association between the stage of CLL/SLL and the severity of renal failure, similar to a previous study [25]. The presence of extrarenal tumoral syndrome was generally associated with interstitial infiltration on the kidney biopsy. An important differential diagnosis for CLL/SLL-related interstitial infiltration is acute interstitial nephritis. The predominance of T cells, and/or the mixed lymphocyte population within interstitial aggregates which respect the normal parenchymal architecture are strong arguments against the diagnosis of CLL/SLL-related infiltration [21].

Interstitial granulomatous nephritis is found in less than 1% of native kidney biopsies, and 6–9% of biopsies revealing interstitial nephritis [26,27]. The most frequent causes of granulomas are infections, drug reactions, and inflammatory disorders, which were systematically excluded in the patients of the present study. In CLL/SLL, the presence of granulomas on kidney biopsy has been reported twice previously [4,28]. Granulomas are a relatively frequent finding in patients with Hodgkin’s lymphoma (14%), and non-Hodgkin’s lymphoma (7%), and are thought to be due to sarcoid reactions against tumor-derived antigens [29]. In this context, the activation of macrophages is typically mediated by T cells, which may explain the lower prevalence in B-cell malignancies. A series of 5 CLL/SLL patients with lymph node and bone marrow granulomas has been reported, which were related to mycobacteria or sarcoidosis, underlining the importance of excluding opportunistic infections in this context [30].

The association of glomerular disease in CLL/SLL has been reported in approximately 50 patients, mainly in case reports and in small cases series [31]. The most frequently reported lesions are MPGN (36%) and membranous nephropathy (19%), the latter frequently presenting with atypical characteristics such as proliferative lesions and monotypic deposits [31]. Less frequently, MCD, focal segmental glomerulosclerosis, amyloidosis, and immunotactoid/microtubular glomerulopathies may be found. A difficult issue in this context is to determine whether or not the glomerular injury is related to the hematologic malignancy. Classical criteria for paraneoplastic glomerulopathies include a chronological relation (GN often revealing the malignancy, or simultaneously diagnosed), a suspected pathophysiological link (such as dysproteinemia with or without cryoglobulinemia, cytokine-altered glomerular permeability, or T-cell dysregulation), and a parallel evolution of the malignancy and the glomerulopathy with specific cytotoxic treatment [32,33]. In our study, 6/15 patients presented a monoclonal dysproteinemia, which is higher than reported in the general population of CLL patients [34,35]. The monoclonal protein secreted by the B-cell clone can either be directly involved in the pathogenesis of the lesions, as is the case in fibrillary glomerulopathy, immunotactoid/microtubular glomerulonephritis, AL amyloidosis, and type I/II cryoglobulinemia, or indirectly, as in cases of MPGN not related to cryoglobulinemia.

MPGN is one of the most frequent kidney diseases described in association with CLL/SLL (Table 8). In our study, 5 patients presented a MPGN, including 2/5 without any detected dysproteinemia. Glomerular lesions associated with cryoglobulinemia are either due to immune complexes or to the direct targeting of native or planted glomerular antigens by the monoclonal cryoglobulin [8,36]. Irrespective of the mechanism leading to cryoglobulin deposition, glomerular infiltration by macrophages is frequent in this context and is believed to play an important pathogenic role [37]. Patient 13 presented a monotypic IgGκ fibrillary GN. The presentation of this patient was unusual since deposits in fibrillary GN are described as polytypic in 90% cases, and hypocomplementemia is rare [38,39]. In addition, fibrillary GN is exceptionally reported in CLL/SLL. Interestingly, Schneider *et al* reported a very similar case, suggesting an important role for complement activation in the pathogenesis of the glomerular lesions in this context [40]. As opposed to the glomerular lesions found in cryoglobulin-related MPGN, immunotactoid/microtubular glomerulonephritis is usually not associated with macrophage infiltration, thrombi or vasculitis-like lesions [38]. The microtubular deposits are monotypic, as in patient 15, their intensity being related to the quantity of the monoclonal component secreted by the B-cell clone [41]. The microtubular organization present in glomerular deposits can also be found in the Golgi apparatus of the malignant lymphocytes [41].

The literature describes 12 previous cases of CLL/SLL-related MCD [42–45]. The pathophysiology of this association is unclear. Both a disturbed pattern of cytokine production by the B clone and a T-cell dysfunction have been proposed. The parallelism between the evolution of the hematological disease and the podocytopathy before and after treatment strongly suggests an immune-mediated link involving the malignant clone.

Immunoglobulin-derived amyloidosis accounts for approximately 85% of amyloidosis diagnosed on kidney biopsy [46]. Few cases of CLL/SLL-related amyloidosis have been reported to date, despite the fact that up to 35% of CLL/SLL patients present abnormal serum free light chain ratios [47–51]. The reason for this theoretical discrepancy may be that the amount of circulating free light chains is usually moderate in CLL/SLL, and that CLL/SLL clones more frequently produce kappa than lambda light chains, lambda light chains typically being more amyloidogenic than kappa [50,51]. In our study one patient presented IgG lambda AHL amyloidosis, which is a particularly rare finding, AHL amyloidosis accounting for less than 5% of Ig-derived renal amyloidosis [46].

This study provides an overview of kidney diseases associated with CLL/SLL requiring biopsy due to decreased eGFR and/or NS. Undoubtedly, the clinical context leading to the indication of histological examination yields a bias in the results of this study. The comparison of our results with those of autopsy series strongly suggests that the prevalence of isolated CLL/SLL-related interstitial infiltration is underestimated in the present study. Inversely, the prevalence of immune-mediated glomerulonephritis is most probably overestimated, compared to the general population of CLL/SLL patients. Yet, we believe that this study provides a valuable overview of the renal lesions expected in CLL/SLL patients with a potential indication for kidney biopsy. Due to the broad spectrum of glomerular and interstitial diseases associated with CLL/SLL, and to the limits of clinicopathological correlations, kidney biopsy remains an essential tool in selected patients with decreased eGFR and/or NS. An important result of this study is that the conclusions of the kidney biopsy led to corticosteroid and/or cytotoxic treatment, and to subsequent favorable renal and hematological responses in a large number of patients.
